# Coexistence of spin ordering on ladders and spin dimer formation in a new-structure-type compound Sr_2_Co_3_S_2_O_3_

**DOI:** 10.1038/srep43767

**Published:** 2017-03-03

**Authors:** Kwing To Lai, Martin Valldor

**Affiliations:** 1 Max Planck Institute for Chemical Physics of Solids, Nöthnitzer Str. 40, 01187 Dresden, Germany

## Abstract

We report on the syntheses and characterizations of single crystalline and polycrystalline Sr_2_Co_3_S_2_O_3_ with a novel crystal structure type. It contains Co–O 2-leg rectangular ladders and necklace ladders. The two ladders share common legs and construct a *hybrid spin ladder*. A rare meridional heteroleptic octahedral coordination is found for the Co^2+^ ions in the 2-leg ladder. Within the necklace ladders, the Co^2+^ ions are in *trans*-octahedral coordination. An antiferromagnetic order is observed at T_N_ ~ 267 K, while a broad maximum in magnetic susceptibility is found below T_N_. This relatively high ordering temperature among Co-based ladder compounds is related to the highly anisotropic *mer*-coordination of the Co^2+^ ions. The *trans*-octahedrally coordinated Co^2+^ ions, on the other hand, corresponds to the possible short-range magnetic correlations through dimers with an effective 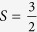. This results in a rare situation that spin ordering and spin dimers coexist down to 2 K.

High-spin Co^2+^ (3*d*^7^) has a spin angular momentum 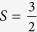 and an orbital angular momentum *L* = 3 according to Hund’s rules. With the cooperation of octahedral crystal field and spin-orbital coupling, the lowest-lying orbital level of Co^2+^ splits into a Kramers doublet, a quartet and a sextet. The Kramers doublet ground state has an effective 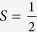 with large Ising-type anisotropy and is separated with the first excited quartet (effective 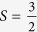) by a energy gap of about 10^2^ K order of magnitude^1^,^2^,^3^,^4^. Hence, low dimensionality in octahedral Co^2+^ compounds can yield novel properties due to the strong quantum fluctuations for 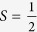 systems. For instance, the quasi one-dimensional (1D) 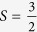 screw chain antiferromagnets *A*Co_2_V_2_O_8_ (*A* = Ba, Sr), which have distorted CoO_6_ octahedra, can be described in terms of a highly anisotropic effective 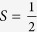 1D *XXZ* model in longitudinal fields^5^,^6^,^7^,^8^,^9^. At high magnetic fields, a field-induced order-to-disorder transition above 1.8 K is observed. The quasi-2D ladder compound Na_2_Co_2_(C_2_O_4_)_3_(H_2_O)_2_ also contains distorted Co^2+^ octahedra. Its magnetic properties can be realized by a 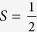 spin-ladder model and show spin-glass behavior^10^,^11^.

Regarding spin-ladder structures, Co-based compounds are relatively rare compared to Fe- and Cu-based compounds. To our knowledge, besides Na_2_Co_2_(C_2_O_4_)_3_(H_2_O)_2_, the available examples are Co(C_8_H_8_O_4_), Co_3_(2,5-*pydc*)_2_(*μ*_3_-OH)_2_(OH_2_)_2_ (*pydc* = pyridinedicarboxylate), Co_7_V_4_O_16_(OH)_2_(H_2_O) and Na_2−*x*_Co_6_(OH)_3_[HPO_4_][H_*x*/3_PO_4_]_3_^11^,^12^,^13^. The properties of Co(C_8_H_8_O_4_) have not been measured, while the rest of them exhibit an antiferromagnetic ordering far below room temperature. Apart from 2-leg ladders, Co(H_2_O){C_5_H_5_N–CH_2_CH(OH)(PO_3_)(PO_3_H)} contains zigzag ladders (see the schematic drawing in Fig. 1a), having frustration within the ladders^14^. According to magnetic susceptibility measurements, it shows no magnetic ordering down to 1.8 K, while a field-induced phase transition is observed at about 1.5 T. Necklace ladders (see Fig. 1b), which can be regarded as 3-leg zigzag ladders, are so far not found in Co-based compounds but in some Cu-based materials like ferrimagnets *A*_3_Cu_3_(PO_4_)_4_ (*A* = Ca, Sr, Pb)^15^.

In this report, the novel ladder-type compound Sr_2_Co_3_S_2_O_3_ is investigated. It demonstrates a new orthorhombic crystal structure type. In Co–O layers, the unique combination of 2-leg rectangular ladders and necklace ladders constructs a *hybrid spin ladder*, a new type of spin ladder. Further, a rare local symmetry of Co^2+^, meridional heteroleptic octahedral coordination by three O^2−^ and three S^2−^ ions, is revealed. With the measurements of magnetic properties and specific heat, an antiferromagnetic transition is found close to room temperature (*T*_*N*_ ~ 267 K) along with a broad maximum in magnetic susceptibility below *T*_*N*_. The broad maximum hints at the possible coexistence of spin ordering and short-range ordering below *T*_*N*_, where the short-range ordering may be formed from dimers with an effective 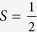.

## Results

### Crystal structure

**Figure 2:**
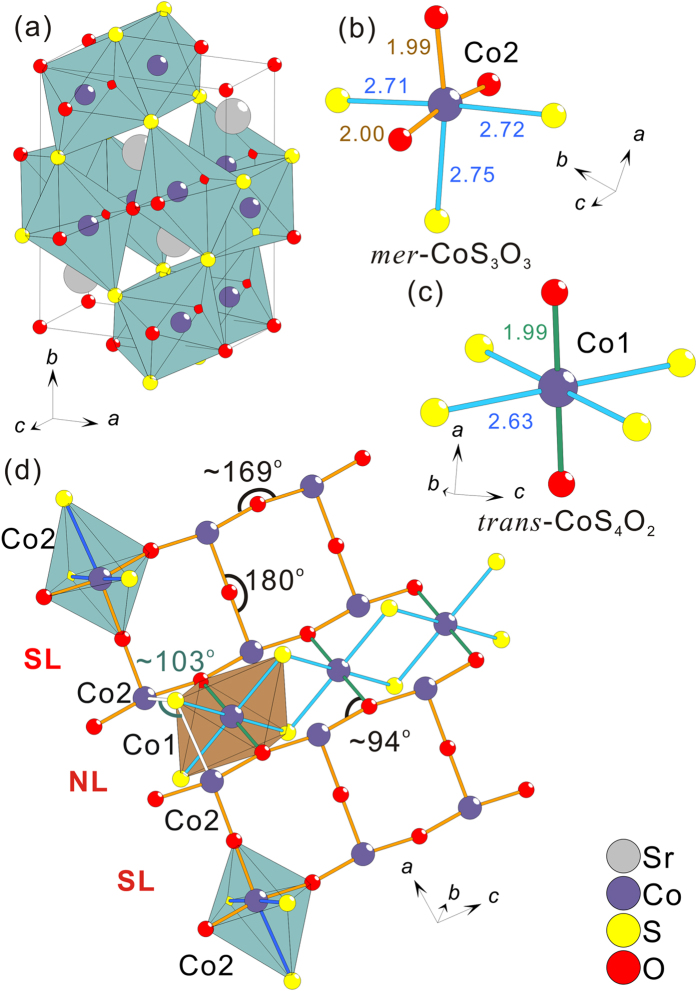
The schematic drawings of crystal structure of Sr_2_Co_3_S_2_O_3_. (**a**) The unit cell. (**b**) The *mer*-CoS_3_O_3_ octahedral coordination in the Co2 site. (**c**) The *trans*-CoS_4_O_2_ octahedral coordination in the Co1 site. The interatomic distances are in Å. (**d**) The Co-O hybrid spin ladder composed of rectangular two-leg ladders (SL) and necklace ladders (NL) alternatively along *ac*-plane.

**Figure 3:**
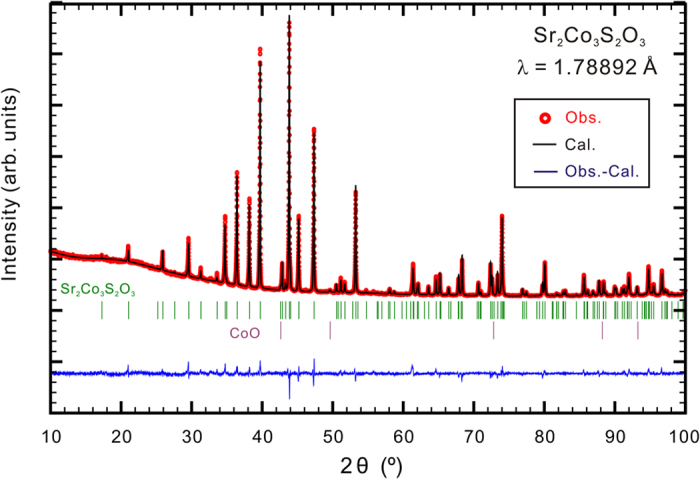
The powder x-ray diffraction pattern of Sr_2_Co_3_S_2_O_3_ at room temperature.

Based on single crystal and powder x-ray diffraction, Sr_2_Co_3_S_2_O_3_ is determined as a new-type orthorhombic structure with a space group of *Pbam*, which is illustrated in Fig. 2a. The single crystal refinement details are presented in Table 1, while the corresponding atomic parameters can be found in Tables 2 and 3. The powder x-ray diffraction data and simulated Rietveld pattern are shown in Fig. 3. The Bragg peaks according to the crystal structure model obtained from the single crystal x-ray diffraction can be well assigned in the powder x-ray diffraction pattern except the peaks from a small amount of CoO impurity (~1%). This is suggestive of a high-quality powder sample. Note that the broad low-angle scattering comes from the sample holder. The elemental analysis from the energy-dispersive x-ray spectroscopy (see Supplementary information) also supports that the sample’s composition is consistent with the nominal composition.

The different aspects of the crystal structure of Sr_2_Co_3_S_2_O_3_ are illustrated in Fig. 2. Due to anion ordering of O and S, a classical structural description starting from a close packing is inappropriate. In a local description, Co^2+^ is exclusively octahedrally coordinated, either by 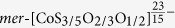 in Co2 sites (Fig. 2b) or by 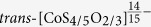 in Co1 sites (Fig. 2c). The former (Co2) constitutes the 2-leg ladder while the latter (Co1) contributes the central spin chain in the necklace ladder (Fig. 2d). The 2-leg ladder and the necklace ladder share the common legs. This unique combination can be referred to as a *hybrid spin ladder*. The Co octahedra build up a three dimensional network by sharing faces and vertices. The interatomic distances are on average: Co–O = 2.0 Å and Co–S = 2.7 Å. These are different from comparable distances in ionic compounds like NaCl-type CoO (Co–O = 2.13 Å)^16^ and NiAs-type CoS (Co–S = 2.34 Å)^17^. The relatively short Co–O and long Co–S distances indicate anomalous bonding behavior, which is accompanied with low local symmetry at the *mer*-coordinated Co site. The shortest Co–Co distance, across face-sharing octahedra, is about 2.9 Å, which is too long for any direct magnetic interactions. Nine-fold coordinated Sr^2+^ ions act as space fillers.

**Figure 4:**
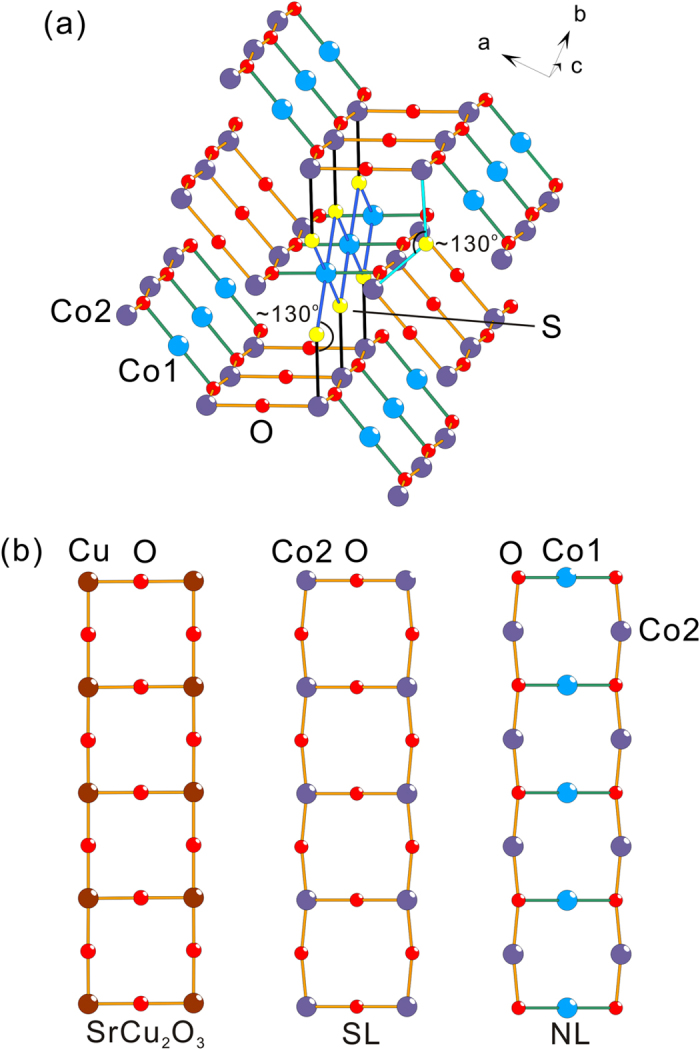
(**a**) The crystal structure of Sr_2_Co_3_S_2_O_3_ with 3 layers of the Co-O hybrid spin ladders. The interlayer bondings with darker color represent the Co1-Co2 interlayer coupling, while that with light color represent the Co2-Co2 interlayer coupling. (**b**) The comparison of ladder lattices among (left) the spin ladder in SrCu_2_O_3_^29^, (middle) the 2-leg rectangular ladder in Sr_2_Co_3_S_2_O_3_ and (right) the necklace ladder in Sr_2_Co_3_S_2_O_3_.

The Co–O–Co angles within the 2-leg ladder are ∠180° for the rungs and ∠~169° for the legs, forming almost ideal rectangular ladders. Meanwhile, the Co–O–Co angles within the necklace ladders are ∠~94°. Between the 2-leg ladders there is no geometrical frustration, i.e. the rungs have the same periodicity in the *ac*-plane, but the *hybrid spin ladder* is frustrated due to intrinsic frustration within the necklace ladders. Three layers of the *hybrid spin ladders*, as displayed in Fig. 4a, reveal the connection perpendicular to the serrated *hybrid* layers. The interlayer couplings are possible via Co–S–Co with ∠~130°.

Before leaving this section, we would like to remark that the oxidation state and spin state of Co ions in Sr_2_Co_3_S_2_O_3_ are expected to be +2 (*d*^7^) and high spin 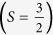 in virtue to the charge balance deduced from the composition and the relatively large Co-octahedra.

### Magnetic properties and specific heat of polycrystals

**Figure 5:**
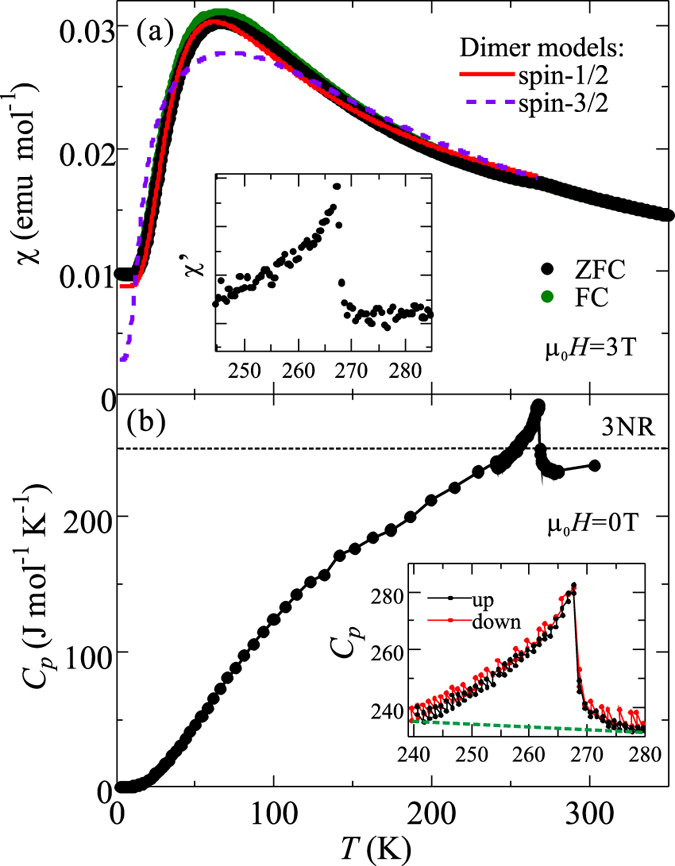
(**a**) The temperature dependence of magnetic susceptibility *χ*(*T*) of polycrystalline Sr_2_Co_3_S_2_O_3_ at 3 T. The dots represent the observed data, while the solid line and the dashed line are the fitted curves according to the 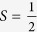 dimer model (Eq. 2) and the 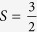 dimer model (Eq. 3), respectively. The inset shows the first derivative of magnetic susceptibility *χ*′(*T*) under zero field cooling. (**b**) The temperature dependence of specific heat *C*_*p*_(*T*) of polycrystalline Sr_2_Co_3_S_2_O_3_ at zero field. The dotted line indicates the Dulong-Petit limit. The inset displays the comparison of the measurements of *C*_*p*_(*T*) with increasing and decreasing temperatures around the transition. The dashed line is an estimate of the phononic contribution.

Figure 5 shows the temperature dependence of magnetic susceptibility *χ*(*T*) and specific heat *C*_*p*_(*T*) of polycrystalline Sr_2_Co_3_S_2_O_3_, respectively. It is obvious in the result from *C*_*p*_(*T*) (Fig. 5b) that there is a *λ*-type peak at *T* ~ 267 K (denoted as *T*_*N*_). Due to the absence of hysteresis comparing the measurements between increasing and decreasing temperature as shown in the inset of Fig. 5b, *T*_*N*_ indicates a second order phase transition. *T*_*N*_ can be also visible in *χ*(*T*) (Fig. 5a) that a small hump is observed around *T*_*N*_. A clearer picture can be seen in the plot of the first derivative of magnetic susceptibility *χ*′(*T*) in the inset in Fig. 5a, which indicates a significant change in *χ*(*T*) at *T*_*N*_. These data suggest that *T*_*N*_ corresponds to a magnetic phase transition. The magnetic entropy released from *T*_*N*_, Δ*S*_*mag*_, is calculated to be about 2.26 J mol^−1^ K^−1^ by using the integral 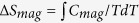, where the magnetic contribution of specific heat *C*_*mag*_ is obtained by subtracting the phononic background in the total specific heat. Since there is lack of non-magnetic isostructural compounds as a reference for the phononic contribution, the background is roughly defined by the specific heat below the dashed line shown in the inset in Fig. 5b. The obtained value is much smaller than the theoretical value *R* ln(2*S* + 1) ~ 11.5 J mol^−1^ K^−1^, where *R* = 8.31 J mol^−1^ K^−1^ is the gas constant. However, considering the purity of the sample, it is safe to assume that this magnetic entropy belongs to the title compound. The small magnetic entropy is typical for low-dimensional systems that Δ*S*_*mag*_ is released in a wide temperature range around the peak. In addition to the rough approximation in our analysis, the phonon contribution is hence easily overestimated and results in the small value of Δ*S*_*mag*_. Therefore, it is not possible to determine from Δ*S*_*mag*_ whether all the Co spins order at *T_N_*.

**Figure 6:**
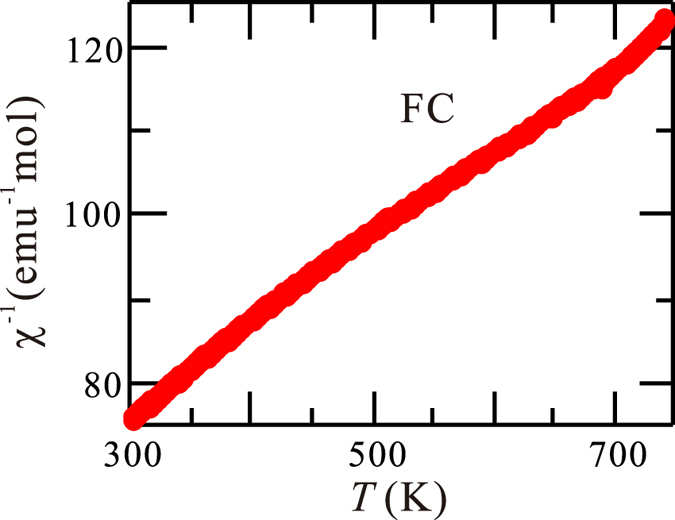
The temperature dependence of inverse magnetic susceptibility *χ*^−1^(*T*) at *T *> 300 K under field cooling.

At *T* < *T*_*N*_, *χ*(*T*) demonstrates a broad maximum around 70 K and approaches to the lowest value but non-zero around 2 K. Meanwhile, there are no anomalies in *C*_*p*_(*T*) ranged from 2 K ≤ *T* < *T*_*N*_, disproving the presence of any further obvious phase transitions below *T*_*N*_. At *T* > *T*_*N*_, *C*_*p*_(*T*) saturates at 3*NR* ~ 250 J mol^−1^ K^−1^ (except the entropy release from the transition), which agrees with the Dulong-Petit limit. Here *N* = 10 is the number of independent atoms in the unit cell. The inverse magnetic susceptibility *χ*^−1^(*T*) at the range *T*_*N*_ < *T* < 750 K, as illustrated in Fig. 6, is not linear, which can be explained by the fact that the first excited orbital levels are thermally populated in this temperature range^18^.

We would like to remark that Sr_2_Co_3_S_2_O_3_ is highly insulating (>5 kΩm) at room temperature, which is expected for a high-spin Co^2+^ oxide.

## Discussion

The uniqueness of the crystal structure of Sr_2_Co_3_S_2_O_3_ are twofold: the meridional (*mer*) heteroleptical octahedral coordination in a magnetic ion and the *hybrid spin ladder. Mer*-octahedral coordinations can be found in some metal organic complexes, with either homoleptic coordination by N in e.g. *mer*-[Co(dien)(NO_2_)_3_] (dien = diethylenetriamine)^19^, [Co(dien)_2_]*X*_3_ · 2H_2_O (*X* = Cl, Br)^20^ and *mer*-[Ni(dien)_2_][SCN]_2_^21^ or heteroleptic coordination by N and O in [Cr(HP_2_O_7_)(NH_3_)_3_(H_2_O)] · 2H_2_O^22^. For non-complex inorganic materials, the *mer*-heteroleptic coordination exists in *M*_2_[Nb_3_O_5_*X*_7_] (*M* = NH_4_, K, Rb, Cs; *X* = Cl, Br) and in La_6_Ti_2_S_8_O_5_, where the octahedra are composed by a Nb^5+^(Ti^4+^) ion surrounding by three O^2−^ and three *X*^−^(S^2−^) ions^23^,^24^,^25^. According to powder x-ray diffraction data and theoretical calculations, *mer*-TaN_3_O_3_ octahedera are also reported in diamagnetic *γ*- and *δ*-TaON^26^,^27^. However, due to the *d*^0^ configuration of the Nb^5+^, Ta^5+^ and Ti^4+^ ions, those compounds should be diamagnetic. Hence, Sr_2_Co_3_S_2_O_3_ is, to our knowledge, the first case where a magnetic ion, here high-spin Co^2+^, is heteroleptically *mer*-octahedrally coordinated in an extended lattice. *Mer*-coordination for *d*^0^, *d*^5^ or *d*^10^ systems is within expectations due to spherical symmetry of these ions. However, it is exceptional to discover such coordination for a *d*^7^ system, because of the uneven electronic occupancy of the *d*-orbitals. Hence, the situation in Sr_2_Co_3_S_2_O_3_ offers the possibility to investigate the effect of a rare crystal field on a magnetic ion.

The *hybrid spin ladder* is the combination of a 2-leg ladder and a necklace ladder. The former is reminiscent of those in cuprates like La_2_CuO_4_, SrCu_2_O_3_ and Sr_2_Cu_3_O_5_^28^,^29^ (Fig. 4b). The necklace ladder can be regarded as the inverse version of the 2-leg ladder (Fig. 4b) and is related to the Cu lattice in *A*_3_Cu_3_(PO_4_)_4_ (*A* = Ca, Sr, Pb)^15^. However, the combination of the 2-leg ladders and the necklace ladders by sharing legs constructs a *hybrid spin ladder*, which is, to our knowledge, a new type of *N*-leg spin ladders. Its uniqueness in competing exchange interactions, including frustration, between magnetic ions can initiate further investigations of novel behaviors through the reproduction of the *hybrid spin ladder*.

In Sr_2_Co_3_S_2_O_3_, *T*_*N*_ is at relatively high temperature compared to other Co-based ladder compounds. This origin can be realized in the *mer*-coordination of the Co2 sites in the 2-leg ladders. Regarding the 2-leg ladders, there are three crucial superexchange interactions: *J*_*rung*_ and *J*_*leg*_ connect Co^2+^ ions via Co–O–Co with about ∠180° along the rungs and the legs in the 2-leg ladders, respectively (see Fig. 2d). *J*_*inter*_ transforms the quasi 1D 2-leg ladders into a 3D network via long Co–S–Co with ∠103–130° (see Figs 2d and 4a). According to the Kanamori-Goodenough rules^30^,^31^, *J*_*rung*_ and *J*_*leg*_ are expected to be antiferromagnetic interactions while *J*_*inter*_ can be antiferromagnetic or ferromagnetic but relatively weak. In addition to the frustration brought from the neighbor necklace ladders, the 2-leg ladders should be able to order antiferromagnetically but not at very high temperatures due to quantum fluctuations. However, as shown in Fig. 2b, the *mer*-CoS_3_O_3_ octahedron has low symmetry with respect to the Co ion. This gives a strongly anisotropic crystal field to the Co ion and thus a preferred orientation for the Co spin, which is referred to the phenomenon called single ion anisotropy. Therefore, although the 2-leg ladders are expected to suppress 3D magnetic orderings because of their low dimensionality, the easy axis for the Co spins in the *mer*-coordination is strong enough to favor the magnetic ordering at higher temperatures.

After the discussion of the spin ordering in the Co2 sites, it naturally comes to the question about the spin ordering of the remaining Co1 sites within the necklace ladders. The observation of the broad maximum in *χ*(*T*) below *T*_*N*_ provides a hint at a rare situation. If all the Co sites order at *T*_*N*_, the broad maximum can stem from the spin canting at the two Co sites. However, this is unlikely due to the following reasons: First, the centrosymmetric space group does not allow for the residual ferromagnetic spin component by spin canting to result in the broad maximum. Second, the Co1 sites are geometrically frustrated. They should be less likely to order at such high *T*_*N*_ unless the spin ordering is somewhat much more energetically favorable. Furthermore, the Co1 sites have *trans*-octahedral coordination with compressed Jahn-Teller distortion (see Fig. 2c). In a rough approximation, the different crystal fields between the two Co sites suggest that they should have different ordering temperatures. This disagrees with the absence of further phase transitions below *T*_*N*_ as seen in *C*_*p*_(*T*). Alternatively, we propose that the Co1 sites could have no long-range ordering down to 2 K. In this framework, the broad maximum corresponds to short-range antiferromagentic correlations for low-dimensional materials. Owing to the mirror plane on the *c*-axis for the centrosymmetric *Pbam* space group, spin dimer formation along the *c*-axis is a possible candidate for the short-range ordering on the Co1 sites.

To prove this argument, the data of *χ*(*T*) below *T*_*N*_ is fitted using the following relation:


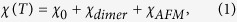


where, the first term *χ*_0_ corresponds to temperature-independent Van-Vleck paramagnetism, diamagnetism and impurity contributions. The second term *χ*_*dimer*_ is the magnetic susceptibility arising from dimers at all the Co1 sites, which correspond to one magnetic Co^2+^ ion per formula. It is modelled by the following equations^32^:


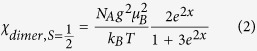


for 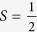 and


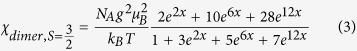


for 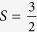, where *N*_*A*_ is Avogadro constant, *μ*_*B*_ is Bohr magneton, *k*_*B*_ is Boltzmann constant, *g* is the Landé g-factor and *x* = *J*/*k*_*B*_*T* with *J* being the nearest-neighbor intradimer exchange constant. The third term *χ*_*AFM*_ represents the magnetic susceptibility contributed from the antiferromagnetic ordering at all the Co2 sites, which correspond to two magnetic Co^2+^ ions per formula. Its temperature dependence is originally presumed according to the mean-field theory that *χ*_*AFM*_ gradually decreases with temperature and reaches 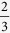 of the value of 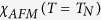 at *T* = 0 K for powder samples. However, it was found during the analysis that the temperature dependence of *χ*_*AFM*_ is much weaker than that of *χ*_*dimer*_, leading to the difficulty of handling multiple free parameters. Hence, to reduce the number of free parameters, this term is thus regarded as temperature independent and refined together with *χ*_0_. Note that the Curie-Weiss contribution arising from the breakdown of dimers due to crystal defects is ignored since no upturn is observed at low temperatures (<10 K).

The fitted curves for 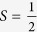 and 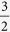 are plotted in Fig. 5a. The curve for 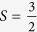 fails to fit the observed curve, rejecting the possibility for 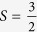 dimers. In contrast, the curve for 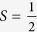 agrees well with the broad maximum of *χ*(*T*). This is suggestive of the existence of quasi-1D dimers with an effective 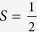 for octahedrally coordinated Co^2+^ ions. The slight discrepancy between the observed and fitted curves may stem from the complex dynamics between the antiferromagnetic ordering and the dimers as well as the oversimplified temperature dependence of *χ*_*AFM*_. The parameters of the fitted curve for 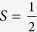 dimers are obtained as *g* = 3.768(8), *J/k*_*B*_ = −49.7(1) K and *χ*_0_ + *χ*_*AFM*_ = 0.00878(6) emu mol^−1^. *g* ≫ 2 supports the anisotropic feature of the effective 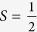 in dimers. The relatively large *J*/*k*_*B*_ suggests that the antiferromagnetic interactions between dimers are strong. The value of 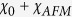 is close to the expected value (~0.008) of the magnetic susceptibility for the antiferromagnetically ordered Co sites (i.e. Co2 sites) at 0 K, agreeing with the assumption made in the fitting analysis.

In order to find out the experimental evidences for the existence of dimers, we have attempted to measure Sr_2_Co_3_S_2_O_3_ by nuclear magnetic resonance (NMR) and electron spin resonance (ESR) techniques. Unfortunately, there is no resolvable signal for further analysis, because the lifetime of nuclear and electronic spin excited states are too short and thus broaden the signal. Note that the existence of spin chains is excluded since no reasonable fits are obtained by using neither the model by Bonner and Fisher^33^ nor the model from Padé approximations by Law *et al*.^34^.

In conclusion, single crystalline and polycrystalline samples of Sr_2_Co_3_S_2_O_3_ were successfully synthesized. The compound is identified as a new-type structure with two interesting features. One is the unique *hybrid spin ladder* consisted of 2-leg ladders and necklace ladders. The other is the rare *mer*-heteroleptical octahedral coordination in magnetic Co^2+^ ions, providing a novel crystal field. Through magnetic property and specific heat measurements of the polycrystalline samples, an antiferromagnetic order forms at *T*_*N*_ ~ 267 K. Such high temperature for *T*_*N*_ is correlated to the highly anisotropic *mer*-coordination of the Co2 sites in the 2-leg ladders, which gives an easy axis for the Co spins to order at higher temperatures than other Co-based spin ladders. Below *T*_*N*_, a broad maximum in *χ*(*T*) is observed along with the absence of further phase transitions. It is suggestive of the short-range magnetic correlations of the Co1 sites within the necklace ladders. The data analysis for the broad maximum proposes the possible coexistence of spin ordering and spin dimers with an effective 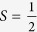 below *T*_*N*_. However, this has to be further confirmed by additional experimental investigations.

## Methods

### Synthesis

Polycrystalline Sr_2_Co_3_S_2_O_3_ was synthesized by solid state reaction using SrO, Co (Alfa Aesar 99.8%), Co_3_O_4_ (Alfa Aesar 99.7%) and S (Alfa Aesar 99.95%) as starting materials. SrO was obtained by heating SrCO_3_ (Aldrich 99.9+%) at 1080 °C overnight at dymanic vacuum (<10^−4^ mbar). The starting materials were mixed to homogeneity inside a dry argon filled glovebox (O_2_, H_2_O < 1 ppm). The mixture was then pressed into pellets and inserted into an alumina crucible. The crucible was inserted into an silica tube which was immediately evacuated to high vacuum (~10^−4^ mbar) and sealed. The sample was annealed at 1050 °C for 20 h. The reaction process was repeated 3 times with intermediate grinding but the annealing time was set to 10 h. Small plate-like single crystals were able to obtain by the similar procedures but were annealed at 1300 °C for 12 h following with cooling to 1050 °C in 96 h. The samples are black and stable in air.

### Sample characterization

Single crystal x-ray diffraction of Sr_2_Co_3_S_2_O_3_ was performed in a Bruker Apex D8 Venture with a Mo-*Kα (λ* = 0.71073 Å) radiation at room temperature. The numerical absorption correction was completed by using XRED (v. 1.07, STOE & Cie GmbH) and X-shape (v. 1.01, STOE & Cie GmbH). The crystal structure was determined and refined by treating the single crystal x-ray diffraction data with the JANA2006 software^35^. The powder x-ray diffraction was carried out in a focusing camera with a Co (*λ* = 1.78892 Å) radiation. The corresponding Rietveld refinement was also performed in JANA2006. Elemental analysis was conducted in an energy-dispersive x-ray spectroscopy (EDX) inside a scanning electron microscope (Philips SEM XL30).

### Measurements of physical properties

The magnetic properties of the polycrystalline samples were measured by a magnetic property measurement system (Quantum Design MPMS XL). For *T* > 350 K, a furnace was inserted for additional heating. The specific heat measurements were performed in a physical property measurement system (Quantum Design PPMS) with the standard non-adiabatic thermal relaxation technique.

## Additional Information

**How to cite this article:** Lai, K. T. and Valldor, M. Coexistence of spin ordering on ladders and spin dimer formation in a new-structure-type compound Sr_2_Co_3_S_2_O_3_. *Sci. Rep.* **7**, 43767; doi: 10.1038/srep43767 (2017).

**Publisher's note:** Springer Nature remains neutral with regard to jurisdictional claims in published maps and institutional affiliations.

## Supplementary Material

Supplementary Information

## Figures and Tables

**Figure 1:**
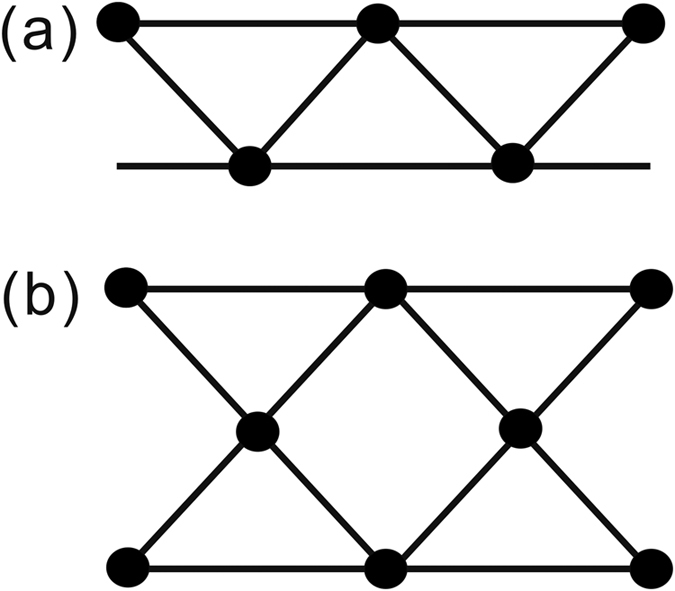
The schematic drawings of (**a**) a zigzag lattice and (**b**) a necklace ladder. The dots represent magnetic ions.

**Table 1:** Details on the single crystal refinement of Sr_2_Co_3_S_2_O_3_ at room temperature.

Empirical formula	Sr_2_Co_3_S_2_O_3_
Crystal system	Orthorhombic
Space group	*Pbam* (No. 55)
*Z*	2
*a* (Å)	7.50285(4)
*b* (Å)	9.79549(5)
*c* (Å)	3.99006(2)
*V* (Å^3^)	293.246(3)
Calculated density (g/cm^3^)	5.2567
Range of data collection	3.42 ≤ 2θ ≤ 65.84
No. of measured reflections	2785
No. of independent reflections	1552
No. of refined parameters	33
*h, k, l* ranges	0 ≤ *h* ≤ 19, 0 ≤ *k* ≤ 25, 0 ≤ *l* ≤ 10
*μ* (mm^−1^)	27.024
*R*(obs), *R*_*w*_(obs)	0.033, 0.090
*R*(all), *R*_*w*_(all)	0.050, 0.14
Goodness of fit	0.70
Final difference density	+0.90/−2.78
Data base - CSD number^a^	431714

^a^Further details of the crystal structure investigations may be obtained from FIZ Karlsruhe, 76344 Eggenstein-Leopoldshafen, Germany (fax: (+49)7247-808-666; e-mail: crysdata@fiz-karlsruhe.de, on quoting the CSD numbers).

**Table 2:** Atomic positions of Sr_2_Co_3_S_2_O_3_.

*Atom*	*Wyckoff*	*x*	*y*	*z*	*U*_*eq*_
Sr1	*4h*	0.09375(4)	0.15762(3)	0.5	0.00617(4)
Co1	*2d*	0	0.5	0.5	0.00822(9)
Co2	*4g*	0.25962(5)	0.41255(4)	0	0.00716(6)
S1	*4g*	0.4051(1)	0.15825(7)	0	0.0066(1)
O1	*2a*	0	0	0	0.0074(5)
O2	*4h*	0.2341(3)	0.4085(2)	0.5	0.0069(3)

**Table 3:** Anisotropic displacement parameters of Sr_2_Co_3_S_2_O_3_.

*Atom*	*U*_11_	*U*_22_	*U*_33_	*U*_12_	*U*_13_	*U*_23_
Sr1	0.00657(8)	0.00687(8)	0.00507(8)	−0.00011(6)	0	0
Co1	0.0055(1)	0.0100(2)	0.0092(2)	0.0022(1)	0	0
Co2	0.0069(1)	0.0110(1)	0.00356(9)	−0.00216(9)	0	0
S1	0.0068(2)	0.0075(2)	0.0056(2)	−0.0003(2)	0	0
O1	0.0054(7)	0.0102(9)	0.0067(8)	0.0011(7)	0	0
O2	0.0068(5)	0.0098(6)	0.0042(5)	0.0002(5)	0	0

## References

[b1] Abragam A. & Bleaney B. Electron Paramagnetic Resonance of Transition Ions (Clarendon, 1970).

[b2] Abragam A. & Pryce M. H. L. The theory of paramagnetic resonance in hydrated cobalt salts. Proc. Roy. Soc. A206, 173–191 (1951).

[b3] Lines M. E. Magnetic properties of CoCl_2_ and NiCl_2_. Phys. Rev. 131, 546 (1963).

[b4] Carlin R. L. Magnetochemistry (Springer, 1986).

[b5] He Z., Taniyama T., Kyomen T. & Itoh M. Field-induced order-disorder transition in the quasi-one-dimensional anisotropic antiferromagnet BaCo_2_V_2_O_8_. Phys. Rev. B 72, 172403 (2005).

[b6] Kimura S. . Field-induced order-disorder transition in antiferromagnetic BaCo_2_V_2_O_8_ driven by a softening of spinon excitation. Phys. Rev. Lett. 99, 087602 (2007). 17930982 10.1103/PhysRevLett.99.087602

[b7] He Z., Taniyama T., Kyomen T. & Itoh M. Antiferromagnetic-paramagnetic transitions in longitudinal and transverse magnetic fields in a SrCo_2_V_2_O_8_ crystal. Phys. Rev. B 73, 212406 (2006).

[b8] Bera A. K., Lake B., Stein W.-D. & Zander S. Magnetic correlations of the quasi-one-dimensional half-integer spin-chain antiferromagnets Sr*M*_2_V_2_O_8_ (*M* = Co, Mn). Phys. Rev. B 89, 094402 (2014).

[b9] Wang Z. . Spinon confinement in the one-dimensional Ising-like antiferromagnet SrCo_2_V_2_O_8_. Phys. Rev. B 91, 140101(R) (2015).

[b10] Honda Z., Katsumata K., Kikkawa A. & Yamada K. Thermodynamic properties in the approach to the quantum critical point of the spin-ladder material Na_2_Co_2_(C_2_O_4_)_3_(H_2_O)_2_. Phys. Rev. Lett. 95, 087204 (2005). 16196897 10.1103/PhysRevLett.95.087204

[b11] Kurmoo M. Magnetic metal-organic frameworks. Chem. Soc. Rev. 38 1353–1379 (2009). 19384442 10.1039/b804757j

[b12] Zhang S.-Y. . Synthesis, crystal structure and magnetic property of a new cobalt(II)vanadate. J. Solid State Chem. 225, 78–82 (2015).

[b13] Yakubovich O. V. . A novel cobalt sodium phosphate hydroxide with the ellenbergerite topology: crystal structure and physical properties. Dalton Trans. 44, 11827 (2015). 26052895 10.1039/c5dt00753d

[b14] Zhang Z.-C., Gao S. & Zheng L.-M. Cobalt diphosphonate with a new double chain structure exhibiting field-induced magnetic transition. Dalton Trans. 4681–4684 (2007). 17940649 10.1039/b709474d

[b15] Yamamoto S. & Ohara J. Low-energy structure of the homometallic intertwining double-chain ferrimagnets *A*_3_Cu_3_(PO_4_)_4_ (*A* = Ca, Sr, Pb) Phys. Rev. B 76, 014409 (2007).

[b16] Jauch W., Reehuis M., Bleif H. J., Kubanek F. & Pattison P. Crystallographic symmetry and magnetic structure of CoO. Phys. Rev. B 64, 052102 (2001).

[b17] Lundqvist D. & Westgren A. Roentgenuntersuchung des Systems Co-S. Z. Anorg. Allg. Chem. 239, 85–85 (1938).

[b18] Burnus T. . Local electronic structure and magnetic properties of LaMn_0.5_Co_0.5_O_3_ studied by x-ray absorption and magnetic circular dichroism spectroscopy. Phys. Rev. B 77, 125124 (2008).

[b19] Churchill M. R., Harris G. M., Inoue T. & Lashewycz A. The meridional isomer of (diethylenetriamine)trinitrocobalt(III), *mer*-[Co(dien)(NO_2_)_3_]. Acta Crystallogr. B37, 933–934 (1981).

[b20] Keene F. R. & Searle G. H. The isomers of the Bis(diethylenetriamine)cobalt(III) ion and a new source of optical activity. Inorg. Chem. 11, 148–156 (1972).

[b21] Mukherjee A. K. . Isomerism in Bis(diethylenetriamine)nickel(ll) thiocyanate: synthesis, solid-state interconversion and x-ray crystallographic study of sym-*fac* and *mer* isomers. J. Chem. Soc., Dalton Trans. 16, 2367–2371 (1994).

[b22] Haromy T. P., Linck C. F., Cleland W. W. & Sundaralingam M. Structures of the meridional and facial isomers of triamminechromium pyrophosphate dihydrate. Acta Crystallogr. C46, 951–957 (1990). 10.1107/s0108270189008401 2393545

[b23] Reusch U. & Schweda E. Preparation and crystal structure of diammonium heptachloropentaoxotriniobate(V) (NH_4_)_2_[Nb_3_O_5_*X*_7_]. Z. Anorg. Allg. Chem. 623, 805–809 (1997).

[b24] Beck J., Bordinhão J. & Kusterer C. On oxohalogeno niobates(V) *M*_2_[Nb_3_O_5_*X*_7_] (*M* = NH_4_, K, Rb, Cs; *X* = Cl, Br) - new members of a compound family with a layered structure. Z. Anorg. Allg. Chem. 633, 757–762 (2007).

[b25] Cody J. A. & Ibers J. A. Synthesis and characterization of the new rare-earth/transition-metal oxysulfides La_6_Ti_2_S_8_O_5_ and La_4_Ti_3_S_4_O_8_. J. Solid State Chem. 114, 406–412 (1995).

[b26] Schilling H. . *γ*-TaON: A metastable polymorph of tantalum oxynitride. Angew. Chem. Int. Ed. 46, 2931–2934 (2007). 10.1002/anie.200604351 17352441

[b27] Lüdtke T. . Synthesis and crystal structure of *δ*-TaON, a metastable polymorph of tantalum oxide nitride. Inorg. Chem. 53, 11691–11698 (2014). 25309986 10.1021/ic501726m

[b28] Longo J. M. & Raccah P. M. The structure of La_2_CuO_4_ and LaSrVO_4_. J. Solid State Chem. 6, 526–531 (1973).

[b29] Dagotto E. & Rice T. M. Surprises on the way from one- to two-dimensional quantum magnets: the ladder materials. Science 271, 618–623 (1996).

[b30] Kanamori J. Superexchange interaction and symmetry properties of electron orbitals. J. Phys. Chem. Solids 10, 87–98 (1959).

[b31] Goodenough J. B. Theory of the role of covalence in the perovskite-type manganites [La, *M*(II)]MnO_3_. Phys. Rev. 100, 564–573 (1955).

[b32] O’Conner C. J. Magnetochemistry-advances in theory and experimentation. Prog. Inorg. Chem. 29, 203–283 (1982).

[b33] Bonner J. C. & Fisher M. E. Linear magnetic chains with anisotropic coupling. Phys. Rev. 135, A640 (1964).

[b34] Law J. M., Benner H. & Kremer R. K. Padé approximations for the magnetic susceptibilities of Heisenberg antiferromagnetic spin chains for various spin values. J. Phys.: Condens. Matter 25, 065601 (2013). 23315241 10.1088/0953-8984/25/6/065601

[b35] Petříček V., Dušek M. & Palatinus L. Crystallographic computing system JANA2006: general features. Z. Kristallogr. 229 **(5)**, 345–352 (2014).

