# Supplementary material for: Coexistence of spin ordering on ladders and spin dimer formation in a new-structure-type compound Sr2Co3S2O3
Source: Sci Rep. 2017 Mar 3;7:43767. doi: 10.1038/srep43767 (PMC5335616; doi:10.1038/srep43767)
Supplement: Supplementary Information [file srep43767-s1.pdf]

Supplementary information for

# Coexistence of spin ordering on ladders and spin dimer formation in a new-structure-type compound $Sr_2Co_3S_2O_3$

Kwing To Lai and Martin Valldor

Max Planck Institute for Chemical Physics of Solids, Nöthnitzer Str. 40, 01187 Dresden, Germany

Email: kt.lai@cpfs.mpg.de

## Elemental analysis from energy dispersive x-ray spectroscopy (EDX)

The elemental analysis of $Sr_2Co_3S_2O_3$ was carried out by measuring 10 points on a single crystal via EDX (Fig. S1). The average results from the 10 measurements show that the ratio of the Sr content to the Co content to the S content is about 2.00(3):3.29(9):1.97(5), as normalized to Sr. This is fairly consistent with the nominal content. Due to highly insulating nature of $Sr_2Co_3S_2O_3$, however, the oxygen content could not be accurately determined.

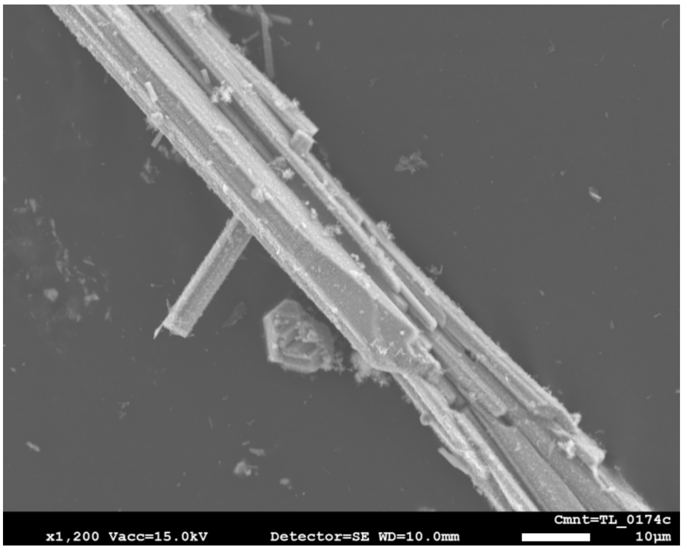


Fig. S1 The SEM image of a single crystal of $Sr_2Co_3S_2O_3$.
